# Bayesian subjectivism and psychosocial attitude toward COVID-19 vaccines

**DOI:** 10.12688/f1000research.121906.1

**Published:** 2022-06-27

**Authors:** Alberto Zatti, Nicoletta Riva

**Affiliations:** 1 Social and Human Sciences, University of Bergamo, Bergamo, Italy, 24129, Italy

**Keywords:** COVID-19 pandemic, decision-making psychological, subjectivism orientation, attitude towards vaccines, attitude change or persuasion, attitude-behavior relations, authoritarianism, Bayesian estimation, citizenship behavior, decision making

## Abstract

**Background**: People resistant to vaccination against the coronavirus disease 2019 (COVID-19) pandemic have been counted in almost all countries worldwide. This anti-scientific subjectivity attitude could be explained by assuming as *background* the individual probability theory originally elaborated by the statistical school of de Finetti. **Methods**: This research method is based on a sample of 613 subjects from European countries who completed a questionnaire on attitudes towards COVID-19 vaccinations. On a six-value scale, a questionnaire investigated knowledge, assessments, degree of confidence, level of fear, anguish, and anger. Some items proposed an imaginary bet on the probability of not getting sick to deepen the possible presence of subjectivist assumptions about pandemics. **Results**: 50.4% were against vaccines and 52.5% against the so-called "Green Pass". Results of t-tests and correlations and *stepwise* regressions indicate that the sample’s reasons for opposing vaccination are related to an ego centred vision of the values that assign minor, if any, confidence to authority. **Conclusions**: This result supports the conclusion that No Vax decisions are more based on subjectivist probabilistic assumptions, thus in line with the significant social trend called "individualism".

## Introduction

In September 2021, one year after the arrival of the first vaccines against coronavirus disease 2019 (COVID-19) infection, a part of the Italian and European population manifested a solid opposition to vaccination. Surveys on the propensity to vaccinate carried out in different countries ( Barello *et al.* 2020; Dratva *et al.* 2021; Mylan & Hardman 2021; Hossain *et al.* 2021) reported significant percentages of individuals wary of vaccination. The health authorities of many countries launched incentive policies for COVID-19 vaccination (see Green Pass in Italy and Europe, but also a lottery in the USA, see Mallow *et al.* 2021). Still, they failed to involve a relatively large percentage of people resistant to vaccination. In Italy, as of October 20, 2021, 9,829,232 citizens of the eligible population group (12 – 99 years old and more) had not yet been vaccinated against the COVID-19 virus (on contrary, at that time, 44,180,669 Italians were partially or completely vaccinated, AA.VV. 2021). Still, in Italy in March 2022, 10% of people who should compulsorily be vaccinated are not.

More recently, some researchers have tried to identify the psychological factors that could explain the anti-vaccine attitude of a part of the population present in each country ( Barello, Palamenghi, Graffigna 2021; Salerno *et al.* 2021; Simione *et al.* 2021; Hughes *et al.* 2021). Research on correlations with personality traits or forms of intellectualism ( Huynh & Senger 2021) and the propensity to populism have made it possible to grasp some psychological variables related to vaccine resistance. However, the results are not entirely evident. With the Big Five test, Murphy and collaborators ( Murphy *et al.* 2021) found that individuals hesitant to vaccinate score lower on personality traits such as agreeableness and conscientiousness. Roccato and Russo (2021) record in a vaccine-hesitant sample little trust in institutions, strong religious beliefs, and a tendency of thought prone to conspiratorial beliefs.

Opposition to COVID-19 vaccination was exacerbated after the request to certify one’s condition with the so-called Green Pass. The legal “pass” called Green Pass was issued following the COVID-19 vaccination. Those in possession of it can go to all public places for all those months declared a pandemic by authorities (full 2021 and early 2022). Subjects not yet vaccinated (from now on indicated for reasons simplification “No/Yes Vax”) and in favour or against the so-call Green Pass is the target of the research. This study aims to prove that the implicit reasoning of the No Vax is better understood within the theoretical framework of subjective probability.

The subjectivist theory of probability ( de Finetti 1959, see also Galavotti 2009 and Savage (1954)) highlights how the prediction on which an individual bases his choices of action depends not only on the probabilities theoretically assignable to an event based on a frequency collected from observation, as evidenced by the theory of causal attribution (see Heider 1958) or on a theoretical probability that can be assumed a priori ( Neyman & Pearson 1933), but on a “calculation” in which the focus is placed on: 1) the intentionality of the subject; 2) his assumptions of value; and 3) his knowledge of the expected probabilities. For example, deciding whether to take the umbrella to leave the house depends on how one sees the weather and if an individual considers getting wet more or less important, his assessment of the “annoyance” of carrying an umbrella, and his desire to show a particular self-image.

Subjective probability theory is a decision theory in a world where uncertainty and degrees of freedom are questionable, primarily because of the specific intentionality of the individual making the prediction. In this regard, Savage (1959) prefers to speak of “personal probability” rather than “subjective probability” to emphasise that the entire complex construct that psychology calls “personality” falls into the evaluations of an individual.

So far, the present research aims to identify the presence of possible individualistic assumptions, let us say structurally ego-centred and anti-authority, in those who are against COVID-19 vaccination and more in those against the Green Pass.

In a previous study (called ‘Anguish and Fears About Attitude Towards Covid-19 Vaccines: Contrasts between Yes and No Vax’), the researchers examined differences in responses on items about fear and anguish about vaccination in the subsample against vaccination vs the subsample in favour of vaccination against COVID-19. In this publication ( Zatti & Riva 2022) authors assumed the hypothesis that No Vax will evidence a kind of anguish (not just a generic “anxiety”) that in psychoanalysis is called “anguish to be invaded” ( Anzieu, 1989). Statistical analysis with Student T-test revealed that the Yes Vax sample has more fear about the COVID-19 pandemic and anguished about social relations and esteem, but the No Vax subsample effectively results in more anguished about the “invasion” of a pharmaceutical substance into their body. In this paper, and using the same dataset, authors focus on another set of item-questions referring to the semantic field simplifying with “how much a person is willing to bet about falling ill by covid”. The formulation of such questions tends to approximate the individualistic probability theory by de Finetti and Savage (1958), which is, this theory, an alternative interpretation of Thomas Bayes’s assumptions. “The role of probability theory in setting inductive logic consists in indicating how the probability assessment relating to future events should change as a result of the result of observed events. From this point of view, inductive logic is substantially reduced to the compound probability theorem or to its slightly more elaborate variant called Bayes’ theorem.” ( de Finetti, 1959, p. 8).

The hypothesis that will therefore be put to the test by this research is that the sample that declares itself No Vax and No Green Pass will have higher values in the questionnaire specifically elaborated on the basis of information collected in the Telegram Chat focused group, here called “ad hoc questionnaire” statements that express the inviolability of individual choices vs the choices of the community, as well as at those statements asking a judgment of public authorities’ decisions.

## Method

### Ethics and consent

Informed consent was obtained from all individual participants included in the study. This, according to art. 13 of EU Regulation 2016/679 and the single country applicable legislation, with particular reference to the Italian Legislative Decree 196/2004 integrated with Decree 101/2018, data collected for the present research guarantees respect for personal privacy sample rights in all senses.

### Participants

A total of 613 individuals from Italy (56.4 %) and other European countries (11.7 % from Poland, 8.5 % from Britain, and a minor percentage from other 15 EU countries, such as Greece, 4.2 %, France, 2.1 %, etc.) undertook this study. Table 1 presents the sample’s characteristics. Respondents were collected online via Prolific services because it provided a special descriptive section on value respondent position in which it is possible to select those people not favourable to vaccinating against COVID-19. With this specific respondent categorization by Prolific, it was possible to recruit a sample enough balanced between Yes and No Vax respondents.

**Table 1.** Sample characteristics.

	Frequency	Percentage
Favourable vaccine	304	49.6
Vaccine contrary	309	50.4
Female	387	63.1
Male	225	36.7
Total	612	99.8
Missing	1	.2
Total	613	100.0

Position toward the Green Pass		
	Frequency	Percentage
I am in favour of the so-called Green Pass	188	30.7
I am against the so-called Green Pass	322	52.5
I do not have a precise judgment on the so-called Green Pass	103	16.8
Total	613	100.0

Country of residence		
	Frequency	Percentage
Italy	346	56.4
Germany	17	2.8
France	13	2.1
Spain	18	2.9
Austria	2	.3
Belgium	3	.5
Czech Republic	1	.2
Denmark	1	.2
Finland	3	.5
Greece	26	4.2
Hungary	17	2.8
Netherlands	12	2.0
Norway	2	.3
Poland	72	11.7
Portugal	27	4.4
Sweden	1	.2
United Kingdom	52	8.5
Total	613	100.0

Sub samples according to crosses judgments on the vaccine and the Green Pass		
	Frequency	Percentage
Yes Vax & Yes Green Pass	182	29.7
No Vax & Yes Green Pass	6	1.0
Yes Vax & No Green Pass	67	10.9
No Vax & No Green Pass	255	41.6
Yes Vax & No Opinion on Green Pass	55	9.0
No Vax & No Opinion on Green Pass	48	7.8
Total	613	100.0

### Procedure

Data were collected online in September 2021. A survey was created and is composed of an ad hoc 92 items (see extended data ( Zatti 2022)). Research questions are original and come from Telegram Chat communication exchange between the researchers and No Vax activists. For this reason, it is not possible to apply reliability criteria, because it is a one-time shot search.

The individuals who responded to the entire questionnaire, including questions on the willingness to bet on the risk of getting sick with COVID-19, were 400. Table 1 shows the distribution of the final research sample (213 subjects did not respond to this last section).

### Measures

Data were collected using an **ad hoc questionnaire.** An original questionnaire was prepared based on the results of an online (via Telegram) focus group with No Vax declared activists. The focus group was conducted in a private chat of no vax activists to which researchers were presented by another activist. An a-synchronal dialectic exchange was conducted with the chat group (it is not possible to count how many people participated). This atypical focus group was conducted during the first two weeks of September 2021. On a six-value scale, the questionnaire proposed statements which the subject had to choose their point of agreement (1 not at all agreed, 6 very much agreed). The questionnaire aimed to collect the subject’s opinions on their social positioning regarding vaccination ( Harré *et al.* 2009).

A series of questions investigated the level of agreement on claims regarding the obligation to vaccinate and the so-called Green Pass (Opinion Index); how much the subject trusts administrative, health, pharmaceutical, and information institutions (Trust Index); and questions on individual choices *versus* public choices (section "Index of Egoic Values vs Society Values" simplified to “Egoic Values Index”). A large section of items required participants to choose, always on a six-level scale, how much they felt fear and how much they felt anger regarding relevant phenomena or events of the COVID-19 vaccination campaign (section "COVID-19 Fear Index" and section "COVID-19 Anger Index"). Another series of questions asked them to choose on the six-level scale how anguished he would feel and how angry he was in general (Section "Anguish Index" and Section "Anger Index in General").

Another section of questions consisted of items in which subjects were asked to choose the probability that they would fall ill with COVID-19 in the future and the “bet” that they would be willing to “gamble” by betting on the fact that they would not get sick in the next six months (section "Subjective Probability Index of getting sick with COVID-19" also called “Subjective Probability Index”). The final index, called “Index pro vs against authority” (summarised in “Authority Index”), asked questions about agreement on the authorities’ policies regarding the pandemic.

Note that tables of correspondences between indexes and items used to build them are reported in the extended data ( Zatti 2022).

Responses were analysed by indexes constituted with the average of single items of the same section. Indexes’ statistical elaboration will be presented on the following pages. The other statistical analysis results can be consulted in the extended data ( Zatti 2022), where it is also possible to read which item follows in each Index.

To evaluate the hypothesis that subjectivist orientation in assessing the attitude towards health policies on the COVID-19 epidemic also involves a position substantially resistant to the decisions coming from authorities, an “Authority Index” was built with the items that asked how much the individuals agreed on the impositions of the institutions.

As reported in the Introduction, the subjectivist theory of probabilities considers that the subjectivist attribution to the occurrence of an event involves the articulation between personal values and knowledge that an individual has of a specific event. To further analyse the subjectivist hypothesis, four new other indexes have been constructed. These “Subjective Indexes” are (comparable items are reported in extended data ( Zatti 2022)): A) Objective Knowledge Index; B) Subjective Knowledge Index; C) Subjectivism-values Index; and D) Authority Index. A strong positive correlation between the “Authority Index” with the indexes “Subjective knowledge subjectivism” (r 0.451, N 530, p 0.00) and “values subjectivism” (r 0.844, N 530, p 0.00) and, also, a strong negative correlation with the “Objective knowledge Index” (r -0.633, N 530, p 0.00) have been reported.

### Statistical analysis

Data were initially analysed through simple descriptive statistics, including means, standard deviations, frequencies and percentages. We then tested for the presence of significant differences between those favourable to COVID-19 vaccination (i.e., the Yes Vax) and those opposed to it (i.e., the No Vax) on all items of the survey through independent samples t-tests, and a p-value ≤ .05 was deemed significant. All analyses were performed with SPSS version 26. Regression analysis and Analysis of Variance were also performed.

## Results

### Subjectivity vs objectivity propensity in No Yes Vax samples

Table 2 shows how the two samples record statistically significant different averages at the t-test only for the items related to the “Egoic Values Index”. Student’s test results are: t = 20.88, df = 611, p < .01 = 99%, confidence interval (Conf. Int.) [1.47, 1.77], in the No Vax sample (M = 4.58, SD = 0.89) compared to the Yes Vax sample (M = 2.96, SD = 1.03).

**Table 2.** Items and Egoic Values Index differences between No and Yes Vax.

	Test t for equality of averages							
	t	Gl	Sign. (two-tailed)	95% difference confidence interval				
				Inferior	Superior		Average	Deviation std.
Egoic Values Index	20.884	611	0.000	1.46716	1.77174	Vaccine Contrary	4.5773	0.88648
						Favourable Vaccine	2.9579	1.02924
The needs of the collective are crushing those of individuals.	12.25	611	0.000	1.16	1.60	Vaccine Contrary	4.14	1.51
						Favourable Vaccine	2.76	1.27
I do not consider it right that duties override the will of an individual	14.11	611	0.000	1.31	1.73	Vaccine Contrary	4.23	1.43
						Favourable Vaccine	2.71	1.23
The values which an individual believes cannot be nullified by the obligations of the authority.	14.75	611	0.000	1.34	1.75	Vaccine Contrary	4.73	1.24
						Favourable Vaccine	3.19	1.34
I want to be myself to determine the values to follow.	14.05	611	0.000	1.30	1.72	Vaccine Contrary	4.93	1.18
						Favourable Vaccine	3.42	1.47
I believe that no person should be obliged by any form of authority.	13.68	611	0.000	1.35	1.80	Vaccine Contrary	4.40	1.49
						Favourable Vaccine	2.83	1.35
Only the individual can decide how to protect his personal health.	20.86	611	0.000	1.94	2.35	Vaccine Contrary	4.86	1.21
						Favourable Vaccine	2.71	1.34

To test the research hypothesis according to which the two Yes Vax and No Vax samples differ in a propensity to assign causal probabilities, such as getting sick with COVID-19, based on different probability attributions (the former in line with the frequentist probability, the latter in line with the subjective or personal probability – Savage, in de Finetti 1959 –) an analysis between items forming the Egoic Values Index was done. Tables 3 and 4 show the correlations recorded by the Yes Vax sample and the No Vax sample between the indexes and the three items that specifically asked for an assessment of the self-attributed probability of getting COVID-19. Statistically significant Pearson correlations are reported at 99% (marked with two asterisks) or 95% (one asterisk).

**Table 3.** Yes Vax significant correlations.

		Egoic Values Index	Trust Index	COVID-19 Fear Index
“I bet I won’t contract the virus in the next six months”	Pearson correlation	.227 ^**^	-.217 ^*^	.173 ^*^
	Sign. (two-tailed)	0.008	0.011	0.045
	N	135	135	135

^*^ Statistically significant Pearson correlations are reported at 95%.

^**^ Statistically significant Pearson correlations are reported at 99%.

**Table 4.** No Vax significant correlations only between items and indexes.

		Egoic Values Index	COVID-19 Rabies Index	Anger Index in General
Subjective Probability Index	Pearson correlation	.246 ^**^	.139 ^*^	.133 ^*^
	Sign. (two-tailed)	0.000	0.024	0.030
	N	265	265	265
"Percentage of the probability of contracting the virus in the future"	Pearson correlation	.145 ^*^	0.119	-0.012
	Sign. (two-tailed)	0.018	0.054	0.841
	N	265	265	265
"I bet I won't contract the virus in the next six months"	Pearson correlation	.207 ^**^	-0.008	0.075
	Sign. (two-tailed)	0.001	0.898	0.223
	N	265	265	265
"Odds of a bet against an opponent who will not contract the virus in the next six months"	Pearson correlation	.161 ^**^	.148 ^*^	.202 ^**^
	Sign. (two-tailed)	0.008	0.016	0.001
	N	265	265	265

^*^ Statistically significant Pearson correlations are reported at 95%.

^**^ Statistically significant Pearson correlations are reported at 99%.

For the Yes Vax sample, there were only three statistically significant correlations of the item “I bet I will not contract the virus in the next six months”: a positive correlation with “Egoic Values Index” r (N 135) = .227, p .008; a negative correlation with the “Trust Index” r (N 135) = -.217, p .011; and a positive correlation with the “COVID-19 Fear Index” r (N 135) = .173, p. 045.

These correlation results were put under a stepwise multiple regression analysis to ascertain the best predictors of the item “I bet not to contract the virus in the future” (v. Table 5). The study indicated a two-variable model, finding that the item “Trust in large pharmaceutical companies” has a Beta weight of .271. This item was inserted first and explained 29% of the variance of the item “I bet not to contract the virus in the future” F = 12.244, p = .001. The item “Fear that the majority of the population determines my behaviours” has a Beta weight of.175. It was inserted second and explained a further 4.8%, F = 4.498, p = .036.

**Table 5.** Stepwise linear regression coefficients Yes Vax sample. ^a^

		Non-standardised coefficients		Standardised coefficients	t	Sign.	95.0% Confidence interval for B		Correlations			Collinearity statistics	
		B	Standard error	Beta			Lower limit	Upper limit	Zero-order	Partial	Part	Tolerance	VIF
1	(Constant)	3.841	.367		10.466	.000	3.115	4.567					
	TRUST LARGE PHARMACEUTICAL COMPANIES	-.384	.110	-.290	-3.499	.001	-.601	-.167	-.290	-.290	-.290	1.000	1.000
2	(Constant)	2.928	.563		5.203	.000	1.815	4.041					
	TRUST LARGE PHARMACEUTICAL COMPANIES	-.358	.109	-.271	-3.292	.001	-.574	-.143	-.290	-.275	-.270	.988	1.012
	FEAR THAT THE MAJORITY OF THE POPULATION WILL DETERMINE MY BEHAVIORS	.216	.102	.175	2.121	.036	.015	.418	.204	.182	.174	.988	1.012

a. Dependent variable: I bet no contract future virus													
Model ^a^ Summary													
Model	R	R-square	Adapted R-square	Std error. of the estimate			Change statistics						
							Modification R-square	Edit F		gl1	gl2	Sign. Edit F	
1	.290 ^to^	.084	.077	1.83168			.084	12.244		1	133	.001	
2	.338	.114	.101	1.80806			.030	4.498		1	132	.036	

^a^ Dependent variable: I bet I don't want to translate the virus in future.

The linear regression shows that the item “Trust towards large pharmaceutical companies” constitutes the primary variable to influence, according to a negative trend, the probability of the Yes Vax sample betting not to contract the COVID-19 virus. This could mean two things: the higher the trust in pharmaceutical companies, the lower the bet of not getting sick, and (or) vice versa.

In the No Vax sample, we find more positive correlations between the index and the items related to risk-taking in a possible bet with the COVID-19 disease. It is relevant that in the No Vax sample, the Egoic Values Index correlates with all three items linked to detecting a tendency to subjective evaluation of future events probability: item “Percentage of the probability of contracting the virus”; item “I bet not to contract the virus in the future”; and item “Betting odds vs opponent”. Finally, in the No Vax sample, for this last item, which evokes a confrontation with an opponent, significant correlations emerge with the indexes of Anger to COVID-19 and Anger in general.

Linear regression was performed to identify which items related to anger can be associated with the item “Bet towards an opponent”, which gave the following results (see Table 6).

**Table 6.** Stepwise linear regression coefficients No Vax sample.

		Non-standardised coefficients		Standardised coefficients	t	Sign.	95. 0% Confidence interval for B		Correlations			Collinearity statistics	
		B	Standard error	Beta			Lower limit	Upper limit	Zero-order	Partial	Part	Tolerance	VIF
1	(Constant)	5. 291	. 519		10. 189	. 000	4. 269	6. 314					
	In general. I can say that I GET ANGRY WHEN LIMITS ARE IMPOSED ON ME	. 405	. 122	. 201	3. 327	. (001)	. 165	. 645	. 201	. 201	. 201	1. 000	1. 000
2	(Constant)	3. 709	. 832		4. 460	. 000	2. 072	5. 347					
	In general. I can say that I GET ANGRY WHEN LIMITS ARE IMPOSED ON ME	. 329	. 125	. 163	2. 640	. (009)	. 084	. 575	. 201	. 161	. 158	. 937	1. 068
	I WANT TO BE MYSELF TO DETERMINE THE VALUES TO FOLLOW	. 375	. 155	. 150	2. 421	. Tel. 016	. 070	. 680	. 191	. 148	. 145	. 937	1. 068
3	(Constant)	2. 904	. 906		3. 207	. (002)	1. 121	4. 687					
	In general. I can say that I GET ANGRY WHEN LIMITS ARE IMPOSED ON ME	. 235	. 131	. 117	1. 794	. Tel. 074	-. 023	. 494	. 201	. 110	. 107	. 835	1. 198
	I WANT TO BE MYSELF TO DETERMINE THE VALUES TO FOLLOW	. 358	. 154	. 143	2. 324	. 021	. 055	. 661	. 191	. 142	. 138	. 934	1. 070
	In general. I can say that I GET ANGRY WHEN SOMEONE TAKES AWAY SOMETHING I CARE ABOUT	. 285	. 132	. 138	2. 167	. Tel. 031	026	. 545	. 198	. 133	. 129	. 875	1. 143

Targeting the item “Odds bet vs opponent” (full text provided as extended data ( Zatti 2022)) and by inserting in the regression all the items that make up the “Egoic Values Index”, the “COVID-19 Anger Index”, and the “Anger Index in general”, the data were subjected to the gradual multiple regression analysis to ascertain which were the best predictors of the item itself. The result was a three-variable model in which, as the first variable, the item “In general I can say that I get angry when limits are imposed on me” had a Beta of .117, which explained 20% of the variance of the target item (F = 11.070, p = 0.001). For the second, the item “I want myself to determine the values to follow”, which has a Beta weight of .143 which explained a further 4% of the variance (F = 5.861, p = 0.016); third, the item “In general I can say that I get angry when someone takes away something I care about” with a Beta of.138 which again explained a 2.1% of the variance of the target item (F = 4.696, p = 0.031).

Targeting now the “Subjectivism Index” and inserting in the regression all the items that create the “Egoic Values Index”, the “COVID-19 Anger Index”, and the “Anger Index in general”, the stepwise multiple regression analysis has been identified as the best predictors of the index itself, a two-variable model. The item “I want to be myself to determine the values to follow” has a Beta weight of .194 which explains the 23.9% the variance of the target index (F = 15.930, p = 0.000) and the item “In general, I can say that I get angry when limits are imposed on me” has a Beta weight of.180 which explained a further 5.7% of the variance of the index (F = 8.7, p = 0.003 – see Table 7).

**Table 7.** Stepwise linear regression coefficients No Vax sample.

		Non-standardized coefficients		Standardized coefficients	t	Sign.	95. 0% Confidence interval for B		Correlations			Collinearity statistics	
		B	Standard error	Beta			Lower limit	Upper limit	Zero-order	Partial	Part	Tolerance	VIF
1	(Constant)	3. 906	. 477		8. 188	. 000	2. 966	4. 845					
	I WANT TO BE MYSELF TO DETERMINE THE VALUES TO FOLLOW	. 369	. 092	. 239	3. 991	. 000	. 187	. 551	. 239	. 239	. 239	1. 000	1. 000
2	(Constant)	3. 358	. 505		6. 644	. 000	2. 363	4. 354					
	I WANT TO BE MYSELF TO DETERMINE THE VALUES TO FOLLOW	. 299	. 094	. 194	3. 176	. 002	. 114	. 484	. 239	. 193	. 187	. 937	1. 068
	In general. I can say that I GET ANGRY WHEN LIMITS ARE IMPOSED ON ME	. 223	. 076	. 180	2. 949	. 003	. 074	. 373	. 229	. 179	. 174	. 937	1. 068

### Cluster analysis crossing subjectivity vs objectivity and against vs propensity to authority

We thus proceeded to extract two categories of individuals with the analysis of the clusters (SPSS Quick Cluster procedure). This statistical analysis highlights two groups (see extended data ( Zatti 2022) and the relative Tables of correspondences between indexes and items used to build them): Subjectivists Against Authority, containing 308 individuals, and Subjectivists in favour of Authority, 222 individuals (see Table 8).

**Table 8.** Multivariate test.

Effect		Value	F	Gl of the hypothesis	Gl error	Sign.
Intercepts	Pillai Track	.988	368.679 ^a^	74.000	323.000	.000
	Lambda di Wilks	.012	368.679 ^a^	74.000	323.000	.000
	Trace of Hotelling	84.465	368.679 ^a^	74.000	323.000	.000
	Larger Roy root	84.465	368.679 ^a^	74.000	323.000	.000
YesNoVacciniEtNoYesAuthorities	Pillai Track	1.238	3.088	222.000	975.000	.000
	Lambda di Wilks	.151	3.845	222.000	969.562	.000
	Trace of Hotelling	3.360	4.869	222.000	965.000	.000
	Larger Roy root	2.657	11.671	74.000	325.000	.000

^a^ Exact statistics.

**Figure 1.**
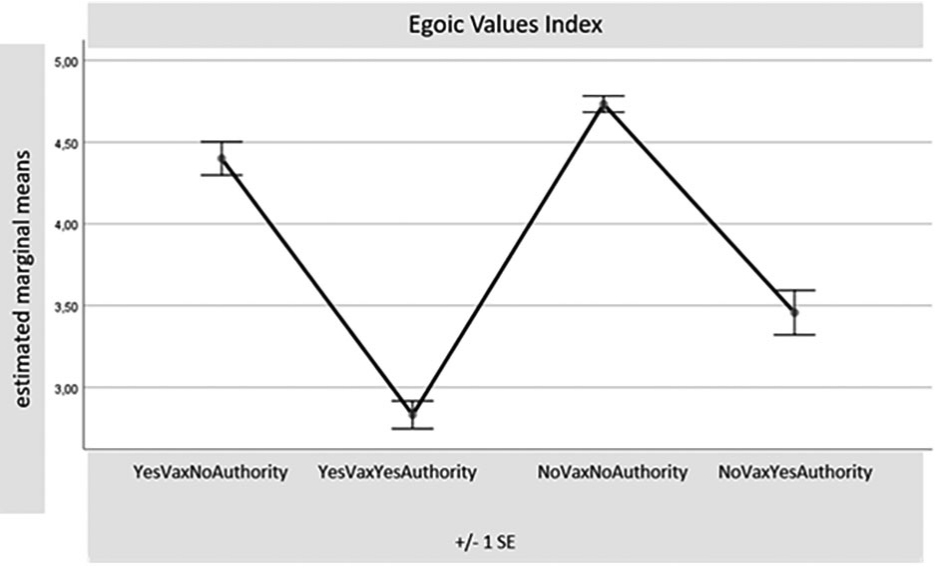
Egoic values index.

**Figure 2.**
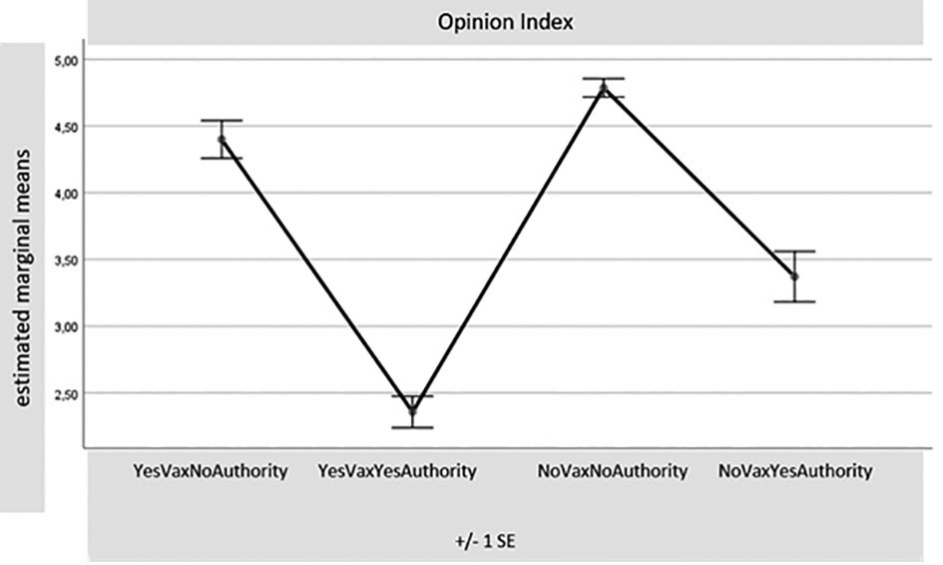
Opinion index.

**Figure 3.**
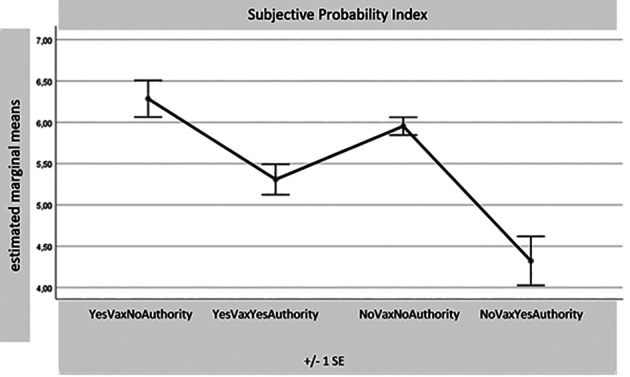
Subjective probability index.

**Figure 4.**
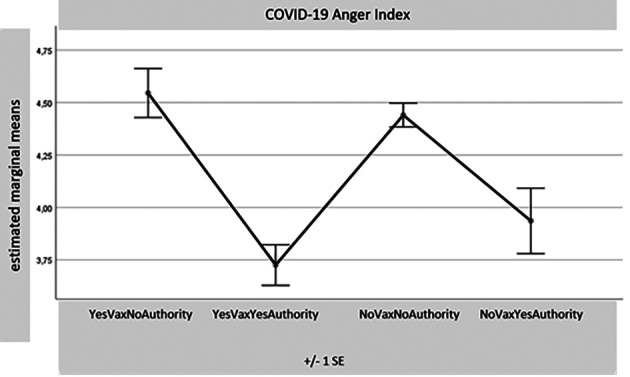
COVID-19 anger index.

Crossing cluster membership with the position for or against vaccination, 59 subjects out of 244 in favour of vaccines (24%) fall within the Subjectivists cluster opposed to the Authority (see table in extended data ( Zatti 2022)). This means that the anti-authority subjectivist position characterises the sample against vaccines and is also present in subjects favouring the vaccine. Vice versa, 37 individuals out of the 286 opposed to the vaccine (13%) are categorised as Subjectivists in favour of Authority. Compared with multivariate analysis, these four sub-samples significantly differ for some significant profiles (see Figures 1- 4). Pillai’s trace (see Table 8) records a significance of 0.000 for all these comparisons. Considering the many differences for each variable found with the MANOVA test between the four samples together, reduced in number (1. Yes Vax No Authority – 55 sub-sample subjects; 2. Yes Vax Yes Authority – 80 SS; 3. No Vax No Authority – 234 SS; and 4. No Vax Yes Authority – 31 SS), only some results of the most relevant differences for this part of the research will be presented. The aim is to indicate potential overlaps between the sub-sample No Vax Yes Authority and the subjects favouring vaccination. For synthesis, only the distributions of the four sub-samples with the four main indexes are presented: Egoic Index, Green Pass Opinion Index, Subjectivism Index, and COVID-19 Anger Index. It seems particularly important to observe the sample which, despite declaring themselves No Vax, turns out to have an attitude accepting Authority.

At the “Egoic Values Index”, the sample under observation (No Vax but Yes Authority) presents an average close to the Yes Vax, thus indicating that the availability of the injunctions of authority lowers the probability of having a self-centred value orientation ( Figure 1). This conclusion is also confirmed by the sample’s position placed under attention records in the “Opinion Index”, below the averages of the two sub-samples against the Authority ( Figure 2).

From Figure 3, it is then noted that the No Vax Yes Authority sample is the one that appears to be much less willing to bet on the probability of not contracting the COVID-19 virus. Finally, it can be seen from Figure 4 how the index of anger towards the limits set by the pandemic is lower precisely for the two sub-samples Yes Vax and favourable toward Authority, as well as in the group that declares themselves No Vax and favourable attitude towards vaccination.

## Discussion

The research highlights how the subjectivist position is associated with evaluating the facts in which individual values filter the personal interpretation of the data of reality. Subjective knowledge, egoic values, and anti-authority are the three factors that support an individualistic approach, in line with what is stated by the theory of personal probability of the Savage and de Finetti school (1959).

The hypothesis of accentuation of subjectivist value assumptions in the No Vax population has been confirmed. The Egoic Values Index and all the items that compose it (see Table 2) are significantly different in the t-test in the means between the sample against the vaccine (No Vax) and the sample in favour of the vaccine (Yes Vax).

The Yes Vax sample records a positive correlation between one of the three items dedicated to the “measurement” of subjectivist probabilistic assumptions. The linear regression analysis shows that it is above all towards pharmaceutical companies that an ambiguous “game” is played because the correlation with the Confidence Index follows a negative trend. As if to say that the less one has confidence in Big Pharma and the more one expects not to get sick in the future, even if, as is known, the correlation could also mean that the more one trusts pharmaceutical companies, the less likely one is prone to bet not to get sick in the future.

On the other hand, the No Vax sample records positive correlations between three indexes (Egoic Values Index, COVID-19 Anger Index, and Anger Index in general) and all the items intended to detect the subjectivist orientation, including the Subjective probability index itself ( Table 4). In the No Vax sample, the analysis of the stepwise multiple regression shows that the “push” to bet not to get sick correlates significantly with some items of the section dedicated to anger in general, such as “I get angry when limits are imposed on me” and “I get angry when something is taken away from me”.

Therefore, the anger emotion seems to be the critical factor of the subjectivist orientation in assessing the probability of not getting sick. The linear regression analysis also adds that the anger of the No Vax individuals seems to increase just when they are confronted with a supporter of the opposite position, a Yes Vax. According to these statistical analysis results, the No Vax subjects’ anger arises from the impositions and the feeling of being defrauded, all accentuated by the opposition, even if only imagined, with an opponent. From the stepwise linear regression analysis, the two main variables related to a subjectivist attitude towards the COVID-19 pandemic are the association between value self-centeredness and anger reaction when limits are set.

The evocation of a subject placed in the out-group ( Tajfel, 1982) seems to induce a growing sense of anger in such individuals. The oppositional relationship particularly emphasises the anger of the No Vax sample: when an antagonist appears (which for the No Vax are the Yes Vax), anger increases.

In our research sample, a certain number of unvaccinated individuals (309 were vaccine contrary but 322 were “against the so-called Green Pass) have also signed they are No Green Pass. A result of the present research shows that some of those favouring vaccination stated they are against the Green Pass itself. This third sub-sample (10.9% of the total) gives a response profile to compare with those of the other two, let’s say, “coherent” samples: the one of Yes Vax and Yes Green Pass and the other of No Vax and No Green Pass. This sample of individuals opposed to vaccinating but sensitive to messages coming from institutions results in being less egoic values-oriented and less reactive to the impositions resulting from the obligation of the Green Pass ( Figures 1 and 2). Moreover, this sub-sample is even the one that, among the four that cross the dimensions Yes/No Vax and Yes/No authority, results in less risk in betting that in the future they will not get sick ( Figure 3). The level of anger resulting from the events of this period of the COVID-19 pandemic is lower than that of the “cousins” No Vax No Authority, even that of the Yes Vax No Authority subjects.

The small number of subjects of the sub-sample No Vax Yes Authority extracted from the analysis of the clusters does not allow a detailed profile of the characteristics of these individuals, even if it does not seem that the gender variables and the level of education are distinctive. A thesis, that for now is only hypothetical, is derived from another set of data referring on a projective tool aiming to describe subjects’ body mental representation (Body Image and Schema Test, Zatti Riva *in press*) and offers partial indications according to which an idealised body image primarily characterises the sub-sample No Vax Yes Authority. In addition, they, like the other sample, Yes Authority Yes Vax, record a higher level of sensitivity to social anguish (i.e. “not being estimated”, “staying alone”) than the two sub samples No Authority. In other words, the No Vax sample who are inclined to authority could be positively influenced by objective communications about the harm of COVID-19 infection due to a potential openness towards the indications coming from the authority.

The fact that a No Vax sample percentage may join the vaccination prevention campaign launched in all countries asks the question of which kind of communication campaign could convince them to vaccinate. It may be that focusing on an idealisation of the vaccine, for instance, on the pioneering, almost heroic, role of the inventors of the M-RNA technology and putting in the background the references to the pharmaceutical industries, would seem to be an applicable indication that the present research can offer to those responsible for the COVID-19 vaccination campaign.

Recalling the classic psychosocial parallelism of Thibaut and Kelley (1959), according to whom the ordinary person evaluates social events implicitly using the laws of statistics (such as covariation), the hypothesis advanced in this study that No Vax individuals do not follow *post hoc* inferences, but they tend to be “subjectivists” from the point of view of the statistical assumptions with which they interpret reality ( de Finetti 1931; Savage 1954) is substantially confirmed. Therefore, the central question on how the No Vax population could be better understood for better communication with them points to turning the theoretical framework to Bayesian statistics, just as the Subjectivist School interpreted Thomas Bayes’ theorems ( de Finetti 1959). The answer to this question is, in fact, the central argument for how public communication can orient social behaviour. To assume that people use *ante hoc* evaluation of social reality, as Serge Moscovici affirm (1988), will implicate that social speech should adopt the scientific language and the symbolic one.

## Data availability

### Underlying data

Open Science Framework: Bayesian subjectivism and psychosocial attitude toward COVID. https://doi.org/10.17605/OSF.IO/FPA3U ( Zatti 2022).

This project contains the following underlying data:

- SpssDataBaseCovidSS613ESSENTIAL2.sav (raw data file, SPSS format)
- BayesianSubjectYesNoVax613SS2 (1).xlsx (raw data file, Excel format)

### Extended data

Open Science Framework: Bayesian subjectivism and psychosocial attitude toward COVID. https://doi.org/10.17605/OSF.IO/FPA3U ( Zatti 2022).

This project contains the following extended data:

- Items related to Indexes calculation.pdf
- Data Key of Questionnaire Items and relative Values.pdf
- Objective and Subjective Knowledge et al Indexes Cluster raw data.pdf
- Subjectivism Article Items Text.pdf
- Tables & Figures.pdf

Data are available under the terms of the Creative Commons Zero “No rights reserved” data waiver (CC0 1.0 Public domain dedication).
