# Supplementary material for: Bayesian subjectivism and psychosocial attitude toward COVID-19 vaccines
Source: F1000Res. 2022 Jun 27;11:703. [Version 1] doi: 10.12688/f1000research.121906.1 (PMC10318375; doi:10.12688/f1000research.121906.1)
Supplement: Peer review history

# Peer review: Reviewer response for version 1

**DOI:** 10.5256/f1000research.133822.r179424

**Published:** 2023-07-03

**Authors:** Igor Sotgiu

**Affiliations:** 1 University of Bergamo, Bergamo, Italy

The authors collected very interesting data about the attitudes towards vaccinations and Green Pass of a large sample of European citizens. My specific comments on the article are reported in the following:

1. I think the article would benefit from expanding the description of the Bayesian theoretical framework. In fact, a reader not trained in statistics might not be familiar with this approach. Otherwise, the authors might also consider to eliminate the word “Bayesian” from the title.
2. In my opinion, the authors could provide further details on the various regression models they estimated to find the best predictors of participants’ belief that they will not contract the virus in the future. Were the potential predictors selected according to any psychological theory? I think that explaining this could significantly improve the readability of the article.
3. Cluster analysis should be mentioned in the Statistical Analysis section of the article. Similar to what I said about the regression analyses, I think that the article would greatly benefit from better explaining the aims of the cluster analysis computed by the authors. In fact, it seems to me that this analysis allowed the authors to test hypotheses which were much more complex than the one they reported in the Introduction (see p. 4, 1 ^st^ paragraph).

Is the work clearly and accurately presented and does it cite the current literature?

Yes

If applicable, is the statistical analysis and its interpretation appropriate?

Partly

Are all the source data underlying the results available to ensure full reproducibility?

Yes

Is the study design appropriate and is the work technically sound?

Yes

Are the conclusions drawn adequately supported by the results?

Yes

Are sufficient details of methods and analysis provided to allow replication by others?

Yes

Reviewer Expertise:

General psychology.

I confirm that I have read this submission and believe that I have an appropriate level of expertise to confirm that it is of an acceptable scientific standard.

# Peer review: Reviewer response for version 1

**DOI:** 10.5256/f1000research.133822.r158411

**Published:** 2023-02-07

**Authors:** Hamid Allahverdipour

**Affiliations:** 1 Department of Health Education and Promotion, Faculty of Health Sciences, Tabriz University of Medical Sciences, Tabriz, Iran

1. In the abstract, the method section is unclear.
2. Introduction is too long.
3. What was the conceptual framework for using "individual probability theory"?
4. In addition, why authors used the bayesian method for analyzing data? For unprofessional who are not familiar with statisticians would be confusing. 
5. I suggest that this paper is suited for review by statisticians.
6. In the method section, more details should be brought about participants and procedures and recruiting of samples.

The following papers may be useful in the introduction and discussion as well:

- Peoples’ attitude toward COVID-19 vaccine, acceptance, and social trust among African and Middle East countries ^1^
- Prioritizing ‘equity’ in COVID-19 vaccine distribution through Global Health Diplomacy ^2^

Is the work clearly and accurately presented and does it cite the current literature?

Yes

If applicable, is the statistical analysis and its interpretation appropriate?

Yes

Are all the source data underlying the results available to ensure full reproducibility?

Yes

Is the study design appropriate and is the work technically sound?

Yes

Are the conclusions drawn adequately supported by the results?

Partly

Are sufficient details of methods and analysis provided to allow replication by others?

Partly

Reviewer Expertise:

Health psychology and behavioral medicine

I confirm that I have read this submission and believe that I have an appropriate level of expertise to confirm that it is of an acceptable scientific standard.

## References

[1] : Peoples' attitude toward COVID-19 vaccine, acceptance, and social trust among African and Middle East countries. *Health Promot Perspect* .2021;11(2) : 10.34172/hpp.2021.21 171-178 10.34172/hpp.2021.21 34195040PMC8233680

[2] : Prioritizing 'equity' in COVID-19 vaccine distribution through Global Health Diplomacy. *Health Promot Perspect* .2021;11(3) : 10.34172/hpp.2021.36 281-287 10.34172/hpp.2021.36 34660222PMC8501485

# Response

**Published:** 2023-02-07

**Authors:** Alberto ZATTI

Dear reviewer, I here answer you in italic at your points: 

 In the abstract, the method section is unclear.

*The research has been based on a questionnaire you can visit on the OSF page linked in the article. It is a psycho-social instrument that asks directly what is a subject's position about a social topic.*

 Introduction is too long.

*I needed to make a longer introduction because the theoretical approach is not so confident.*

 What was the conceptual framework for using "individual probability theory"?

*De Finetti and Savage are two eminent representatives of the statistical school called "subjective probability". This statistical school was, at the same time, a theoretical and a technical school. In my article, I assumed the theoretical approach to probability, that is, that people use their own subjective judges to attribute a certain probability to an event and/or to a behaviour.*

 In addition, why did the authors use the bayesian method for analyzing data? For unprofessional who are not familiar with statisticians would be confusing. 

*The article statistical elaboration are not realized with the bayesian statistic method. The De Finetti and Savage approach interpret Thomas Bayes theorem's in the subjective manner. This is because the bayesian statistic is based on the effective frequencies ov an event, not on the theoretical probability of it. But, in the article, the statistic used is traditional. Bayesian subjectivity, as you read in the title, is the way we used to stress the subjectivity approach of the study.*

 I suggest that this paper is suited for review by statisticians.

*I hope to have made clearer with the above answer that statistic used in the article is very classic, let's say.*

 In the method section, more details should be brought about participants and procedures and recruiting of samples.

*As reported in the article, the standard recruiting of a psycho-social sample is via the professional services of private societies as Prolific. It was just a random recruiting onto the specific database of a panel of subjects prone or not to vaccination.*
